# Map of thrombogenesis in viral infections and viral-driven tumours

**DOI:** 10.1007/s12672-022-00610-1

**Published:** 2023-01-08

**Authors:** Beatriz Vieira Neto, Valéria Tavares, Joana M. O. Santos, Fátima Cerqueira, Deolinda Pereira, Rui Medeiros

**Affiliations:** 1 Molecular Oncology and Viral Pathology Group, Research Center of IPO Porto (CI-IPOP)/ Pathology and Laboratory Medicine Dep., Clinical Pathology SV/ RISE@CI-IPOP (Health Research Network), Portuguese Oncology Institute of Porto (IPO Porto)/Porto Comprehensive Cancer Center (Porto.CCC), 4200-072 Porto, Portugal; 2 FMUP, Faculty of Medicine, University of Porto, 4200-072 Porto, Portugal; 3 ICBAS, Abel Salazar Institute for the Biomedical Sciences, Rua de Jorge Viterbo Ferreira, 228, 4050-313 Porto, Portugal; 4 Oncology Department, Portuguese Institute of Oncology of Porto (IPOP), 4200-072 Porto, Portugal; 5 FP-I3ID, FP-ENAS, FP-BHS, University Fernando Pessoa, Praça 9 de Abril, 349, 4249-004 Porto, Portugal; 6 Faculty of Health Sciences, University Fernando Pessoa, Rua Carlos da Maia, 296, 4200-150 Porto, Portugal; 7 Research Department, Portuguese League Against Cancer (NRNorte), 4200-172 Porto, Portugal

**Keywords:** Viruses, Immune system, Neoplasms, Thrombosis, Thromboprophylaxis

## Abstract

Viruses are pathogenic agents responsible for approximately 10% of all human cancers and significantly contribute to the global cancer burden. Until now, eight viruses have been associated with the development of a broad range of malignancies, including solid and haematological tumours. Besides triggering and promoting oncogenesis, viral infections often go hand-in-hand with haemostatic changes, representing a potential risk factor for venous thromboembolism (VTE). Conversely, VTE is a cardiovascular condition that is particularly common among oncological patients, with a detrimental impact on patient prognosis. Despite an association between viral infections and coagulopathies, it is unclear whether viral-driven tumours have a different incidence and prognosis pattern of thromboembolism compared to non-viral-induced tumours. Thus, this review aims to analyse the existing evidence concerning the association of viruses and viral tumours with the occurrence of VTE. Except for hepatitis C virus (HCV) and human immunodeficiency virus (HIV) infection, which are associated with a high risk of VTE, little evidence exists concerning the thrombogenic potential associated with oncoviruses. As for tumours that can be induced by oncoviruses, four levels of VTE risk are observed, with hepatocellular carcinoma (HCC) and gastric carcinoma (GC) associated with the highest risk and nasopharyngeal carcinoma (NPC) associated with the lowest risk. Unfortunately, the incidence of cancer-related VTE according to tumour aetiology is unknown. Given the negative impact of VTE in oncological patients, research is required to better understand the mechanisms underlying blood hypercoagulability in viral-driven tumours to improve VTE management and prognosis assessment in patients diagnosed with these tumours.

## Introduction

### Viral infections and the immune system: link to carcinogenesis

**Fig. 1:**
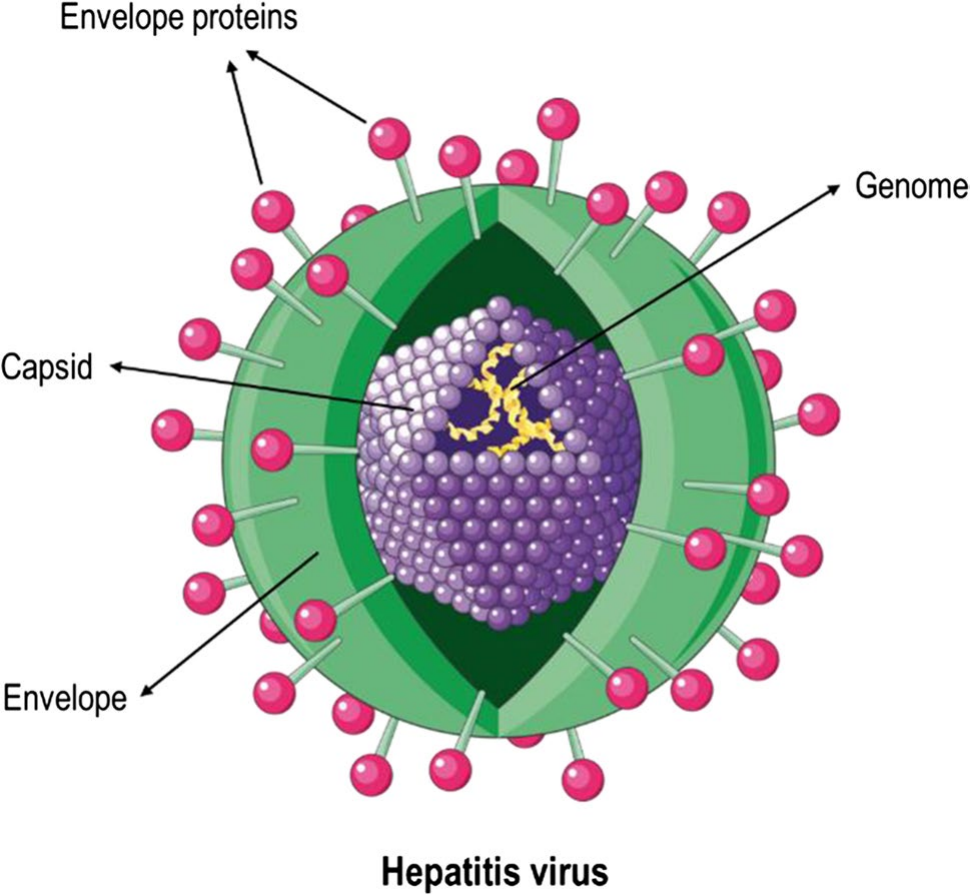
Structure of an enveloped virus. Viruses have three main structural components, namely the viral genome, the protective genome-surrounding capsid and, occasionally, an outer layer of a membrane with proteins and lipids known as the envelope. The figure includes pictures from Servier Medical Art. Servier Medical Art by Servier is licensed under a Creative Commons Attribution 3.0 Unported License (https://creativecommons.org/licenses/by/3.0/).

Viruses are versatile and life-threatening pathogens known for being the aetiological agents of several infectious diseases and malignant neoplasms in animals [1, 2]. Despite their common properties and a large range of potential hosts, several viral families exist and can be distinguished based on morphology, genome structure, chemical composition and mode of viral replication [3]. Viral particles generally have two main structural components: their genome and the surrounding capsid. Additionally, they can also have an outmost lipid bilayer, known as the envelope, that closely surrounds the protective shell of the core genome (Fig. 1). The genetic material can be either a single-stranded or double-stranded DNA or RNA, which determines the strategy used for viral replication [3].

The host immune system has two components that work together to prevent viral invasion and progression and to optimize the response to pathogens [4]. The first component, known as the innate immune response, operates as the first line of defence through the detection of pathogen-associated molecular patterns (PAMPs), which in the setting of viral infections, consist of viral genomes and/or proteins. The recognition of these PAMPs by pattern recognition receptors (PPRs), which are host germline-encoded specialised receptors, triggers a downstream signalling that induces the production of pro-inflammatory cytokines, particularly type I interferons (IFNs), to respond against the viral infection. This unspecific but quick response is essential for the subsequent adaptive immune response. In opposition to the innate immune response, this second layer of protection is highly specific and effective due to the activation of T and B cells with the consequent development of immune memory against a particular pathogen, in this case, a virus [5, 6].

Unfortunately, viruses can negatively affect the host’s immune system’s capacity to either control or fight the infection, allowing a viral infection to become persistent. When such occurs, homeostatic cellular processes responsible for maintaining host genome integrity can be disrupted, causing genomic instability in the infected cells, which might lead to viral-mediated carcinogenesis [7, 8]. Indeed, multiple viruses constitute oncogenic agents, inducing malignant cell transformation and further cancer development, particularly when additional factors are present. Although the mechanisms underlying carcinogenesis may vary according to the viral agent, shared signalling pathways are recognized [9]. In many cases, viral proteins are the main drivers of malignant transformation as they interact and ultimately inhibit tumour suppressor proteins. As a result, cells infected with oncoviruses may eventually present genetic alterations that trigger carcinogenesis, which, combined with their unrestricted proliferation, promotes the accumulation of tumour cells [9–12].

Interestingly, roughly 10% of human cancers are attributable to persistent viral infection [13]. However, it should be noted that an oncogenic viral infection itself does not imply cancer initiation; instead, it increases cancer risk in infected individuals since the host and environmental factors contribute to viral carcinogenesis [9, 12, 14]. Over the years, research in tumour virology has allowed the identification of several viruses capable of causing cancer direct and indirectly in humans (Table 1).

**Table 1:** Oncoviruses and status of evidence concerning thrombosis

Viral family	Virus	Viral genome	Nº of viral infections per year	Associated tumours	Key tumour-promoting mechanism	No of cancer cases per year (2020)^a^	Evidence of an association between the virus and VTE
*Hepadnaviridae*	HBV [48]	Partially dsDNA	~ 1.5 million [59]	HCC [57]	Genetic damage caused by immune-mediated hepatic inflammation, oxidative stress induction, genomic instability due to HBV DNA integration in the host genome, epigenetic alterations (i.e., DNA methylation) and deregulation of microRNAs expression [57]	HCC: 905 677	Yes
*Flaviviridae*	HCV [48]	ssRNA	~ 1.5 million [60]	HCC and NHL [167, 168]	Inflammation, deregulation of cell proliferation, apoptosis, vesicular trafficking, oxidative stress and gene expression [169]	HCC: 905 677 NHL: 544 352	Yes
*Papillomaviridae*	HPV [126]	dsDNA	14 million in the US [170]	CC, anogenital and oropharyngeal cancers [171]	Inactivation of p53 and Rb leading to cell cycle deregulation and apoptosis suppression [171]	CC : 604 127	Scarce
*Herpesviridae*	EBV [172]	dsDNA	NA	HL [88] NPC [88] GC [88] NHL: BL, DLBCL and ENKTL-NT [88]	EBV products (e.g., latent membrane proteins (LMPs), nuclear antigens (EBNAs), untranslated RNAs and microRNAs) modulate cancer progression by promoting epithelial-mesenchymal transition, cell motility, invasiveness angiogenesis and metastasis [12]	NHL: 544 352 HL: 83 087 NPC: 133 354 GC: 1 089 103	Scarce
	KSHV [138]	dsDNA	NA	KS [173]	Angiogenesis and Inflammation, cell migration and invasion [141, 173]	KS: 34 270	Scarce
*Retroviridae*	HIV [107]	ssRNA	~ 1.5 million [108]	AIDS-defining cancers: KS, HL, NHL [109]	Chronic inflammation and cytokine deregulation, immunosuppression [109]	KS: 19 560	Yes
	HTLV-1 [147]	ssRNA	NA	ATLL [174]	Upregulation of cellular genes that mediate lymphocyte proliferation and resistance to apoptosis (inactivation of p53). Genetic instability mediated by the generation of reactive oxygen species and dysfunctional DNA repair processes [147]	NA	Scarce
*Polyomaviridae*	MCPyV [175]	dsDNA	NA	MCC [176]	Sequestration and inactivation of Rb leading to cell cycle deregulation, inactivation of apoptosis and defective DNA repair mechanism [176, 177]	NA ~ 2000 in US [178]	Scarce

*ATLL* Adult T-cell leukaemia/lymphoma, *BL* Burkitt lymphoma, *CC* Cervical cancer, *DLBCL* Diffuse large B-cell lymphoma, *dsDNA* Double strand DNA, *EBV* Epstein-Barr virus, *ENKTL-NT* Extranodal NK/T lymphoma, nasal type, *GC* Gastric cancer, *HBV* Hepatitis B virus, *HCC* Hepatocellular carcinoma, *HCV* Hepatitis C virus, *HIV* Human Immunodeficiency virus, *HL* Hodgkin lymphoma, *HPV* Human Papillomavirus, *HTLV-1* Human T-lymphotropic virus type 1, *KS* Kaposi sarcoma, *KSHV* Kaposi’s sarcoma-associated herpesvirus, *MCC* Merkel cell carcinoma, *MCPyV* Merkel cell polyomavirus, *NHL* non-Hodgkin lymphoma, *NPC* Nasopharyngeal carcinoma, *NA* Unknown, *ssRNA* Single strand RNA

^a^According to GLOBOCAN [62]. Exceptions were referenced in the table

### Viral infections and the immune system: link to thrombogenesis

Haemostasis is a biological process triggered following vascular damage to curtail blood loss, provide a barrier to infection and restore vascular integrity, allowing continuous blood circulation throughout the body [15]. To do so, the haemostatic system has a highly regulated interplay between three main components, namely platelets, plasma proteins and the vasculature [16–18]. Under physiological conditions, there is a permanent and counteracting force assuring the equilibrium between pro-coagulant and anti-coagulant mechanisms to prevent haemorrhage (state of blood hypo-coagulation) and thrombosis (state of blood hyper-coagulation). However, certain pathological conditions, mainly concerning the endothelial cell’s integrity and/or the expression levels of plasma proteins, may disrupt the haemostatic equilibrium towards either thrombotic or haemorrhagic events [17–19].

**Fig. 2:**
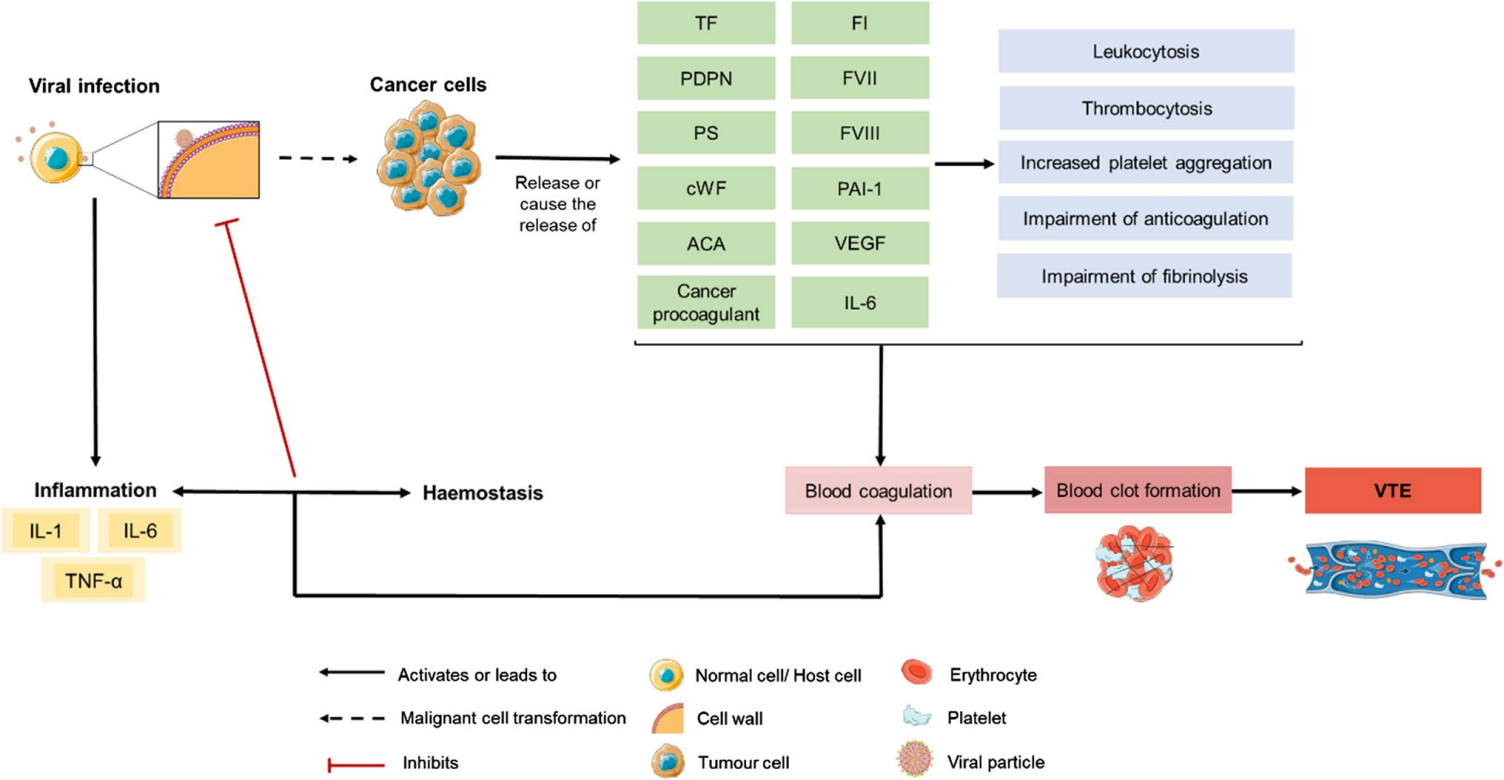
Interplay between viral infection, tumorigenesis and VTE development. Tumour cells are known to cause a pro-thrombotic cascade that may lead to VTE development. Likewise, viruses, well-known oncogenic agents, can also trigger thrombogenesis through the establishment of a pro-inflammatory state that can disrupt the haemostatic balance, given the close interplay between immunity and coagulation. *ACA* antiphospholipid anticardiolipin antibodies, *cWF* von Willebrand factor, *FI* fibrinogen, *FVII* coagulation factor VII, *FVIII* factor VIII, *IL-1* Interleukin 1, *IL-6* Interleukin 6, *PAI-1* plasminogen activator inhibitor-1, *PDPN* podoplanin, *PS* phosphatidylserine, *TF* tissue factor, *TNF-α* tumour necrosis factor-alpha, *VEGF* vascular endothelial growth factor, *VTE* venous thromboembolism. The figure includes pictures from Servier Medical Art. Servier Medical Art by Servier is licensed under a Creative Commons Attribution 3.0 Unported License (https://creativecommons.org/licenses/by/3.0/)

Haemostasis and immunity are two interdependent and complementary pathways whose co-evolution has strengthened the host’s defence against a wide range of pathogens [20]. The undeniable bidirectional relationship is proven by the influence each system has on one another, with inflammation triggering haemostasis, which in turn affects inflammatory activity [4, 21]. This coordinated loop created between inflammation and haemostasis acts to fight against infections and maintain tissue integrity [22]. Indeed, several inflammatory mediators influence haemostatic mechanisms, namely interleukin 1 (IL-1), interleukin 6 (IL-6) and tumour necrosis factor-alpha (TNF-α), which are synthesized during innate immune response upon infection [20, 22, 23]. Given the complex interplay between haemostatic and immune systems, with both systems constantly influencing each other (Fig. 2), a viral infection constitutes a risk factor for thrombosis as it induces a pro-inflammatory state with an underlying pro-thrombotic cascade [4, 20, 22, 23].

### Trousseau syndrome: what about viral-driven tumours?

Thrombosis is a common cardiovascular and life-threatening disease that arises from the generation of a thrombus in the arterial or venous circulation, often followed by the generation of an embolus, which can lead to the reduction or even blockage of the blood flow [24]. Characterized by complex pathophysiology, this cardiovascular disorder encompasses arterial and venous thromboembolism (VTE), each with different aetiology and treatment approaches, the latter being more common among cancer patients [25, 26].

Venous thrombogenesis includes deep vein thrombosis (DVT), its primal manifestation, and pulmonary embolism (PE), a repercussion of DVT in 95% of the cases. Like other common diseases, VTE pathogenesis involves both acquired and genetic factors, whose interplay significantly disturbs the haemostatic equilibrium towards thrombosis [27–29]. One important acquired risk factor for VTE development is cancer.

Armand Trousseau first observed the association between malignant disease and thromboembolism in the nineteenth century by realizing the major medical impact this haemostatic disorder had on cancer patients’ prognosis [30]. In fact, the blood hypercoagulation state in the context of cancer followed by the diagnosis of VTE, known as the Trousseau syndrome, is nowadays remarkably well-recognized and, aside from the malignant neoplasia itself, is the second most frequent cause of death among these patients [31, 32]. Several studies have demonstrated a growing incidence of cancer-related thrombosis, which can be explained by patient- (e.g., advanced age), cancer biology- (e.g., primary tumour site, histology, size and stage) and cancer treatment-related factors (e.g., surgery, chemotherapy and radiotherapy) [28, 30, 33, 34]. Beyond VTE itself, the negative impact of the disease on cancer patient prognosis can also be attributed to the contribution of haemostatic mechanisms in cancer progression and aggressiveness. On the one hand, cancer cells release several components, alone or within microvesicles, that are known to trigger a thrombo-inflammation state, including pro-clotting proteins (e.g., coagulation factors VII (FVII) and VIII (FVIII), cancer pro-coagulant, phosphatidylserine (PS), podoplanin (PDPN) and tissue factor (TF)), inflammatory cytokines (e.g., IL-6, which induces megakaryopoiesis), fibrinolytic regulators (e.g., plasminogen activator inhibitor-1 (PAI-1)) and pro-angiogenic factors (e.g., vascular endothelial growth factor (VEGF)) (Fig. 2). Furthermore, cancer cells can also stimulate other cells (platelets, endothelial cells and leucocytes) to produce and release pro-thrombotic components (Fig. 2) [35–40]. As a result, venous thrombogenesis may occur alongside thrombocytosis, leucocytosis and anaemia [36, 41, 42]. On the other hand, changes in the haemostatic system towards thrombogenesis favour tumour neo-angiogenesis, immune evasion and metastasis, contributing to cancer progression in several ways [35, 43]. This interplay between tumour cells and haemostatic components results in a loop between tumour progression and thrombogenesis that contributes to the development of Trousseau syndrome and cancer aggressiveness [24, 27].

While a two-way relationship between cancer and thromboembolic events is well-recognized, few studies have pinpointed an association between thrombogenesis and viral-driven tumours [27]. The solid association of viral infections with both thrombosis and carcinogenesis, as well as an increased risk for thrombogenesis in the setting of malignancy, raises the question of whether patients with viral tumours may have a different pattern of thromboembolic events and whether the occurrence of these events may have a distinct prognostic value in these patients compared to individuals with non-viral induced tumours. Indeed, oncogenic viruses may sustain chronic infections, with the production of viral particles, which may last for the whole life of the patient [44]. As viral persistency and/or latency are compatible with tumorigenesis, oncological patients may carry the virus in a dormant stage, which can further be reactivated, which is the case, for instance, for infected hepatitis B virus (HBV) patients [44, 45]. Thus, it is biological that the chronic inflammation associated with the presence of the virus and/or viral particles may influence the development of thrombotic events among cancer patients. In this context, the primary purpose of this review was to gather and analyse the evidence concerning the association of viruses and viral-driven tumours with the occurrence of thromboembolic events, and to explore how these events relate to one another.

## Viral carcinogenesis and VTE incidence

Eight oncoviruses are currently recognized as being associated with a variety of tumours, namely HBV, hepatitis C virus (HCV), Epstein-Barr virus (EBV), human papillomavirus (HPV), Kaposi’s sarcoma-associated herpesvirus (KSHV), human T-cell lymphotropic virus type 1 (HTLV-1), human immunodeficiency virus (HIV) and Merkel cell polyomavirus (MCPyV) [46, 47]. Concerning an association with VTE incidence, two groups of viruses and related tumours can be identified based on the current data, one with strong and the other with scarce evidence, as described in Table 1. The former includes HBV, HCV, EBV and HIV, while the latter encompasses HPV, KSHV, HTLV-1 and MCPyV. To be noted, the incidence of cancer-related VTE according to tumour aetiology (for instance, viral carcinogenesis versus chemical carcinogenesis) is not reported in the literature, at least to the best of our knowledge.

### Oncoviruses with a reported association with VTE

#### Hepatitis B and C virus: hepatocellular carcinoma and non-hodgkin lymphomas

HBV is a well-known member of the *Hepadnaviridae* family, while HCV belongs to the *Flaviviridae* family [48]. Despite being both highly tissue and host- specific, infecting preferentially human hepatocytes, their genomic organization and replication strategy is quite different, as well as their mode of transmission. Specifically, while HBV is a partially double-stranded DNA virus commonly spread through exposure to blood and body fluids (saliva, semen, etc.), HCV is a single-stranded RNA virus that mainly spreads through contact with infected blood [49–51].

The HBV and HVC viruses have been associated with one of the most common and serious infections worldwide, known as chronic liver infection, which is currently considered a major health problem due to its life-threatening potential. This condition refers to the long-term viral hepatitis (meaning viral-driven inflammation of the liver) that results from the progression of the initial acute stage of infection and is responsible for the development of cirrhosis (fibrosis of the liver causing impaired organ function), a major risk factor for liver cancer [52–54]. In 2019, around 296 million people worldwide were HBV seropositive, with the vast majority of the cases reported in the WHO Western Pacific and African Regions, while approximately 58 million people were chronically infected with HCV [55–60].

Both viruses can cause malignancy in humans, particularly liver cancer, a highly prevailing and fatal tumour [49, 58]. Liver cancer is the seventh most incident and the fourth leading cause of cancer deaths worldwide, with hepatocellular carcinoma (HCC) accounting for approximately 90% of the diagnosed cases [7, 61, 62]. In 2020, liver cancer was associated with around 900 600 newly diagnosed cases and 830 180 deaths [62]. As expected, this malignant disease is particularly common in Eastern Asia and Sub-Saharan Africa, where HBV and HCV chronic infections are most prevalent. As previously stated, HBV and HCV infections with the subsequent development of cirrhosis represent the leading aetiological factor for HCC, accounting for approximately 80% of the cases. Several additional risk factors are thought to also play a role in the disease development, including cigarette smoking, alcohol consumption and other comorbidities [49, 55, 57, 58, 63, 64]. Although still unknown, evidence suggests that, in addition to liver cancer, HBV and HCV are also significantly linked to non-Hodgkin lymphomas (NHL), a heterogeneous group of common hematologic malignancies accounting for 90% of the total lymphoma incidences [65]. This group encompasses a broad range of disorders that can be either highly aggressive or indolent and are characterized by the transformation and proliferation of lymphoid cells in the lymph nodes, gastrointestinal tract, bone marrow, liver and spleen [65, 66]. In 2019, NHL was particularly frequent in Eastern Asia, with 544 352 cases and 259 793 deaths globally reported in 2020 [62, 65]. The NHL fraction attributable to HCV varies geographically, ranging from 1 to 2% to more than 10%, due to different HCV prevalence across countries. Besides, HBV infection is associated with a 2.50-fold increase in NHL risk [48, 67].

The impact of HBV and HCV chronic infections on the development of thromboembolic events (regardless of cancer status), is a subject of discussion among researchers worldwide. The increasing interest is underpinned by the influence these viruses have on the haemostatic system [68]. Despite several studies reporting a high presence of VTE in individuals with HCV infection, the direct association between both pathologies remains unclear, and data are contrasting [69–71]. Nevertheless, a recent meta-analysis and retrospective studies show that HCV-infected patients present a significantly high risk of developing venous thrombosis [70]. As for HBV chronic infection, it might be a thrombotic risk factor, however, it is not yet entirely explicit whether it causes VTE or portal vein thrombosis, a condition that among some populations is suggested to be mainly caused by HBV [69].

It remains uncertain whether HBV and HCV cause thrombotic events or whether other confounders may be present [69]. If the former is true, several biological pathways may be at the centre of this phenomenon. One possible explanation could be the impaired blood flow/vasculopathy deriving from the inflammatory state triggered by HBV and HCV chronic infections, specifically the immune-mediated hepatic inflammation associated with the unstable cytokine secretion by hepatic cells [72]. As it is well-known, chronic liver disease is frequently associated with complex alterations in the haemostatic system, most likely due to the pivotal role liver has in the synthesis and clearance of activated coagulation factors and platelet production through thrombopoietin (TPO) synthesis [73]. Specifically, the haemostatic disturbance can be triggered by changes in platelet counts with the onset of thrombocytopenia (low platelet count) during the stage of chronic liver disease, as well as a decreased synthesis of anti-coagulant proteins (protein C, protein S and antithrombin III) and increased synthesis of pro-coagulant ones (for instance, von Willebrand factor (cWF)) by liver cells [70, 72, 74–77]. Thrombocytopenia is a complex and multifactorial pathology that, among other underlying causes, can result from impaired TPO production due to hepatic damage during chronic liver disease [77]. Although uncommon, thrombocytopenia is associated with thrombogenesis, an event particularly highlighted during COVID-19 vaccination [78]. Oxidative stress induction has also been suggested to explain VTE in viral hepatitis patients, with a previous meta-analysis and case reports reporting an association between viral hepatitis and antiphospholipid antibodies (aPL) positivity. This condition is thought to be strongly correlated with venous and arterial thrombosis [68, 69, 79].

**Fig. 3:**
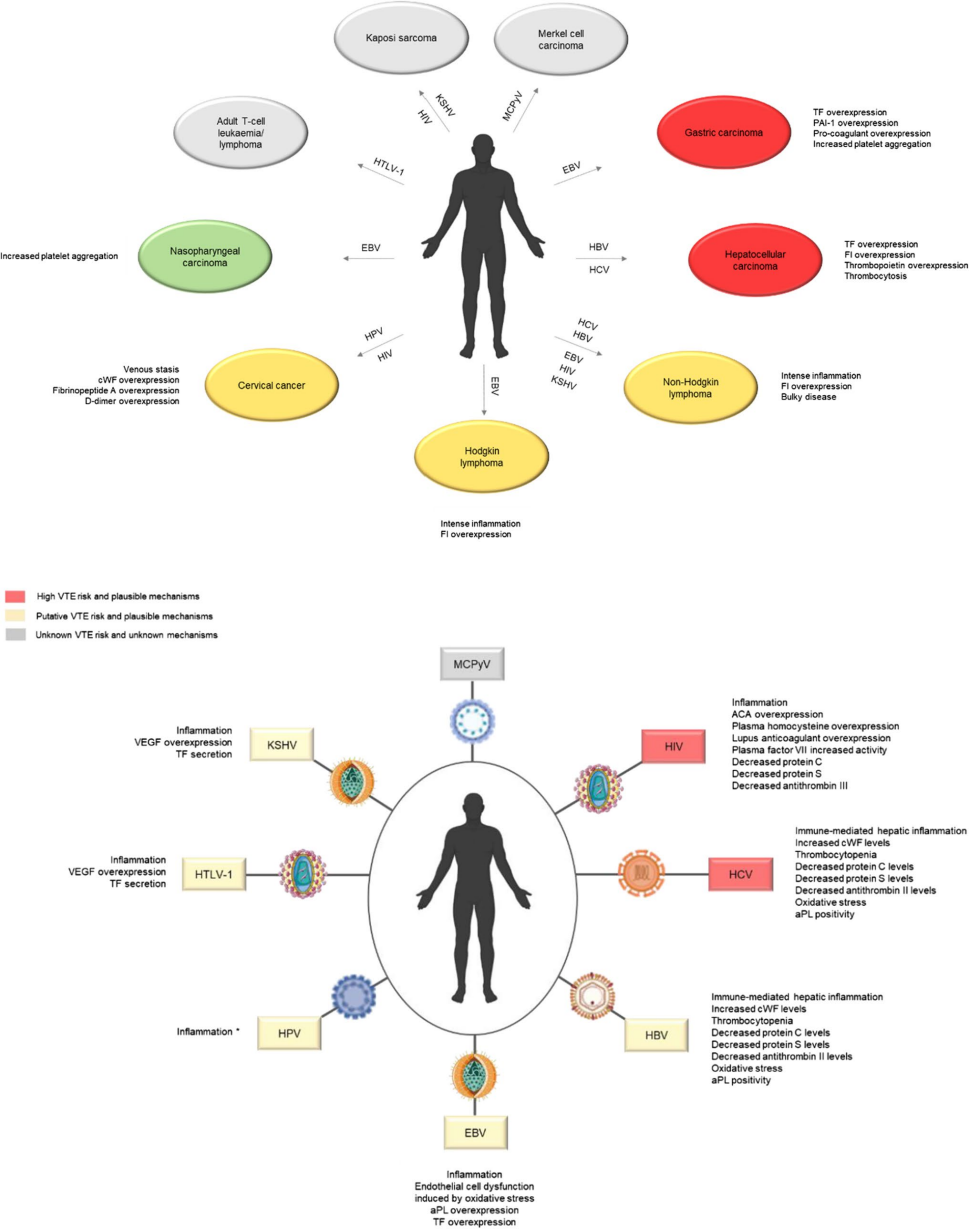
**a **Map of viral-driven tumours and their thrombosis-associated risk and putative underlying mechanisms.** b **Map of oncoviruses and their thrombosis-associated risk and putative underlying mechanisms. *ACA* antiphospholipid anticardiolipin antibodies, *aPL* antiphospholipid antibodies, *cWF* von Willebrand factor, *EBV* Epstein-Barr virus, *FI* fibrinogen, *HBV* hepatitis B virus, *HCV* hepatitis C virus, *HIV* human immunodeficiency virus, *HPV* human papillomavirus, *HTLV-1* human T-lymphotropic virus type 1, *KSHV* Kaposi sarcoma-associated herpesvirus, *MCPyV* Merkel cell polyomavirus, *PAI-1* plasminogen activator inhibitor-1, *TF* tissue factor, *VEGF* vascular endothelial growth factor. * Inflammation due to HPV vaccination. The figure includes pictures from Servier Medical Art. Servier Medical Art by Servier is licensed under a Creative Commons Attribution 3.0 Unported License  (https://creativecommons.org/licenses/by/3.0/)

As for the pattern of development of thromboembolic events in HBV and HCV-associated tumours, starting with HCC patients, the risk of developing VTE is highly increased (high-risk group; Fig. 3) [54]. In fact, according to a retrospective study by Wang et al. [80], VTE was identified in approximately 6% of 270 HCC patients. Indeed, thrombosis in these patients may be prompted by both cancer and liver cirrhosis (prevalent in around 85–95% of HCC patients) due to their perturbation of the haemostatic balance. Although the biological processes underlying the blood hypercoagulability in the setting of HCC remain unclear, recent studies have suggested several explanatory mechanisms, including overexpression of TF (also known as coagulation factor III) and fibrinogen (coagulation factor I), as well as thrombocytosis (increased platelet count) [54, 81]. Despite being rare in this context, the latter might result from the overproduction of TPO by hepatic tumour cells, contributing to the development of VTE [82]. As for NHL patients, the risk of VTE is comparable to that observed among patients with pancreatic and ovarian cancers (intermediate-risk group; Fig. 3). Recent studies have suggested, however, a risk heterogeneity among this group of malignancies, with diffuse large B-cell lymphoma (DLBCL) being the histotype with a greater risk of venous thrombogenesis, a phenomenon potentially explained by the presence of an intense inflammatory response, bulky disease and increased levels of fibrinogen [83, 84]. As previously mentioned, in opposition to HCC, only a small fraction of NHL cases is potentially caused by HBV and HCV infection, and the incidence of cancer-related VTE according to tumour aetiology is currently unexplored. As such, it is unclear to what extent the high risk of VTE development among NHL patients is associated with these oncoviruses.

Overall, studies performed throughout the last decade support a hepatitis-related pro-thrombotic state (intermediate/high-risk group; Fig. 3). Given the impact both viral infections might have on platelet and coagulation factor levels, with these components playing a central role in haemostasis and inflammation, as well as in cancer growth and metastasis, these events together could easily contribute to blood hypercoagulability in hepatitis patients, ultimately promoting thrombosis, and simultaneously cancer progression [70, 74]. In addition, the viruses are linked to tumours with an intermediate to high risk of VTE (NHL and HCC). Whether the prothrombogenic potential in these tumours can be partially attributed to HBV and HCV needs to be explored.

#### Epstein-Barr virus: non-hodgkin lymphoma, Hodgkin lymphoma, nasopharyngeal carcinoma and gastric carcinoma

The EBV, also known as Human Herpesvirus type 4 (HHV-4), is a double-stranded DNA gamma-herpesvirus whose infection is commonly associated with multiple human diseases [85]. According to epidemiological studies, the EBV infection’s worldwide prevalence is remarkably high, with over 90% of the adult population currently infected [86].

Over the years, several studies have identified an association between EBV infection and a broad range, yet a small fraction of cancers [87, 88]. Among many well-recognized malignant human diseases, infected individuals are at increased risk for NHL (e.g., Burkitt lymphoma (BL)), Hodgkin lymphoma (HL), gastric carcinoma (GC) and nasopharyngeal carcinoma (NPC), in addition to other less common related diseases [89]. The geographical distribution patterns of these tumours are strongly influenced by EBV-related, genetic and environmental factors, and predictably, the highest incidence rates are found in relatively low- and middle-income regions, particularly East Africa and Eastern and South-Eastern Asia [64]. As for the worldwide fraction in 2020, it was estimated that 239 700 to 357 900 new cases and 137 900 to 208 700 deaths of attributable-EBV cancers, particularly NHL, HL, NPC and GC [88, 90]. Previously, EBV has been detected in approximately 52% of all lymphomas (of which 54.5% were NHL and 40.9% were HL cases) [91]. Furthermore, around 10% and 80% of all GC and NPC cases are associated with EBV infection, respectively [92, 93]. When dismissing divergencies related to the host and cancer biology, there is a clear and prevailing pattern in the carcinogenic process across all EBV-related tumours, which involves the suppression of TSGs with the subsequent deregulation of apoptosis, cell cycle and immune signalling pathways [12, 92, 94].

Data is still lacking regarding the impact of EBV infection on the development of thromboembolic events [69]. Despite being seldom reported mostly in immunocompromised patients, according to a few case-reports studies, this cardiovascular disease has also been identified in immunocompetent patients suffering from an acute EBV infection, which contributed to the worsening of their clinical outcome [69, 87, 95–98]. Interestingly, some of these patients were found to have elevated levels of aPL, a variable identified as favouring blood clot formation, as previously stated [95, 96]. Although poorly understood, to the best of our knowledge, venous thrombosis in EBV patients might be related to oxidative-inducing endothelial cell injury and inflammation, with the consequent secretion of TF [69, 87, 95, 96].

As for the pattern of thromboembolic events in EBV-associated tumours, beyond NHL (associated with intermediate risk as previously mentioned), HL and GC patients also exhibit a significantly high thrombotic risk. For instance, in a study by Kirkizlar et al. [89], VTE was detected in 20.7% of the HL patients (intermediate-risk group; Fig. 3) and contributed to increased morbidity and mortality. The inflammatory state with the subsequent increasing fibrinogen levels was indicated as the presumable trigger of thrombosis in the setting of HL. As for GC, this malignancy is well recognized to be associated with one of the highest incidences of cancer-related venous thrombosis (high-risk group; Fig. 3). According to Tetzlaff et al. [99] study in GC patients, the reported incidence of VTE ranges from approximately 5 to 25% depending on the cancer stage, which is detrimental to patients’ survival [100]. Among these patients, the underlying pro-thrombotic mechanisms include elevated levels of TF, PAI-1 and cancer pro-coagulant and tumour-induced platelet aggregation, among other determinants [99, 101]. Conversely, recent studies have shown that VTE incidence among NPC patients is relatively low (4.3%). This is in line with what is observed in head and neck tumours, which are usually included in the low VTE risk group (low-risk group; Fig. 3) compared to other solid tumours [102, 103]. Researchers appoint to increased platelet aggregation and a blood hypercoagulable state in NPC patients, which might be key factors in triggering thrombosis [104–106]. However, in these patients, these mechanisms seem to be only relevant in the context of thrombosis related to the use of central catheters rather than thrombogenesis induced by the tumour biology itself [103, 106].

Despite being seemingly benign and harmless, EBV infection is a possible trigger of many disorders, including cancer and VTE. Regarding thrombogenesis, the mechanisms are not fully understood. Nevertheless, the EBV-associated tumours, except for NPC, are linked to an intermediate/high risk of thrombosis, which might have a connection with the pro-inflammatory environment created by EBV infection alongside the tumour’s interplay with haemostatic components.

#### Human immunodeficiency virus (HIV): Kaposi sarcoma, non-hodgkin lymphoma and cervical cancer

HIV is a member of the *Retroviridae* family that targets immune cells and whose infection, despite manageable, continues to be a major public health problem [107, 108]. In 2020, approximately 1.5 and 40 million people worldwide acquired and were living with HIV, respectively, with central African regions being the most affected areas [108]. Interestingly, the highest prevalence of this virus in low- and middle-income countries correlates with the high burden of virally associated cancers in these regions [108, 109]. Although there is no known cure for HIV-infected patients, they can benefit from treatment regimens such as highly active antiretroviral therapy (HAART). In fact, in the absence of this treatment, HIV infection can easily progress to acquired immunodeficiency syndrome (AIDS), the most advanced and severe stage of the viral infection, which is often followed by the development of opportunistic infections, including oncoviruses due to the patients’ weakened immune system, which explains a high incidence of subsequent malignant diseases [108, 110].

Most HIV-associated cancers are caused by HBV, HCV, EBV, KSHV, HPV and MCPyV. Tumours including KS, cervical cancer (CC) and NHL, lung cancer, HL, HCC and head and neck tumours, among others, are found to be particularly common among patients with HIV infection. Among those, KS, CC and NHL are defined as AIDS-defining tumours, meaning they are more likely to occur in patients with AIDS [109, 111, 112]. The former is a vascular neoplasm whose etiologic agent is KSHV, considered the dominant malignancy among AIDS patients [113]. In 2020, there were over 34 000 newly diagnosed cases and 15 086 deaths, of which around 70% were HIV-associated [62, 114]. On the other hand, although with a gradually decreasing incidence due to screening and vaccination, CC is one of the most common gynaecological neoplasia diagnosed in women worldwide, and virtually all cases are attributed to HPV [115]. In 2020, more than 604 000 cases and 341 000 CC deaths were reported globally, with 4.9% of the cases being associated with HIV infection in 2018 [62, 116]. As for HIV-attributable NHL cases, according to Coté et al. [117], in the United States, among approximately 50 000 patients with AIDS, 4.3% had NHL.

It is also well-known that HIV carriers are commonly diagnosed with VTE, presenting a two- to ten-fold increase in the disease risk of developing compared to the general population. This risk varies according to AIDS and non-AIDS status. The HIV-related blood hypercoagulability and the consequent high VTE prevalence greatly depend on the interplay between traditional thrombotic risk factors and HIV-related factors, as well as HIV treatment-related factors (e.g. HAART) [118, 119]. Indeed, it has been noted that HAART, specifically those including protease inhibitors (PIs), plays a key role in the predisposition for thromboembolic events among HIV-infected patients by causing metabolic and somatic alterations with known pro-thrombotic effects. Underlying this phenomenon may be TNF-α and PAI-1 levels, which seem to be elevated in some of these patients, suggesting thrombo-inflammation during HIV infection and possible deregulation of the fibrinolytic process [119, 120]. A hypothesis is that the pro-inflammatory environment can either promote the expression of pro-coagulant factors (e.g., antiphospholipid anticardiolipin antibodies (ACA), homocysteine, lupus anti-coagulant and coagulation factor VII) and downregulate the synthesis of the anti-coagulant ones (e.g., antithrombin III, protein C and protein S), as well as induce the deregulation of fibrinolysis and endothelial cell dysfunction [118, 120]. More specifically, viral proteins and the associated inflammatory mediators interact with endothelial cells triggering downstream pathways, which may favour both leukocyte and platelet activation, as well as their adhesion to the endothelium, generating thrombo-inflammation, therefore predicting VTE [118, 120]. However, it is relevant to mention that even though increased levels of ACA can be found, evidence regarding its correlation with VTE in this particular group of patients is lacking [119].

As for the pattern of thromboembolic events in AIDS-defining tumours, besides NHL, which was previously described, a study conducted by Jacobson et al. [120] found 23% of HIV-positive and AIDS patients diagnosed with VTE, specifically DVT, had KS as exclusive tumour comorbidity. González-López et al. [121] suggested that thrombotic events might play an important role in the pathogenesis of KS, however, the mechanisms underlying the linkage between KS and VTE, to the best of our knowledge, have not been described (unknown-risk group; Fig. 3). As for VTE incidence in CC patients, previous studies have reported an incidence ranging between 0 and 34% (intermediate-risk group; Fig. 3) [122]. In two retrospective studies by Jacobson et al. [123] and Matsuo et al. [122], a VTE incidence of 11.7.% and a clear difference in survival time between thrombotic and non-thrombotic CC patients were observed, as well as an incidence of 12.3%, respectively. Overall, thromboembolism incidence in CC patients is considered significant (intermediate-risk group) and has a negative impact. Although little information exists, the tumour-related risk is postulated to derive from venous compression and stasis due to CC location rather than tumour biology [124]. However, increased count of platelets and white blood cells, as well as low haemoglobin, have been suggested to be risk factors for increased VTE risk among these individuals. Furthermore, increased levels of cWF, fibrinopeptide A and D-dimer, all related to coagulation activation, have been reported in CC patients with advanced-stage disease, which might explain blood hypercoagulability in these patients [24, 122, 124]. Indeed, in metastatic CC, the risk of developing VTE is comparable to the one reported in advanced-stage ovarian cancer, a highly thrombogenic gynaecological malignancy [122]. Nevertheless, the influence of tumour location (venous compression) and treatment-related determinants in the expression of these factors cannot be dismissed.

In summary, although further investigation is required for clarification purposes, several factors may form the primary triggers for thrombosis in HIV carriers. Chronic inflammation is the primary triggering event in HIV patients towards carcinogenesis, which also seems to constitute a potential stimulus towards thrombosis. Remarkably, AIDS-defining tumours present distinct VTE incidences and can be divided into two groups according to VTE incidence: intermediate-risk (CC/NHL) and unknown association with VTE (KS). In general, NHL and CC patients share the same pro-thrombotic alterations that ultimately lead to VTE onset, whereas KS-thrombotic mechanisms are poorly explored [109].

### Viruses with unknown association with VTE

#### Human papillomavirus (HPV): cervical cancer

HPV is a member of the *Papillomaviridae* family, whose persistent infection is highly associated with the development of cancer, and other less severe conditions, such as anogenital and cutaneous warts [125, 126]. According to Centers for Disease Control and Prevention (CDC), around 43 million infections with HPV were estimated in 2018 and 13 million new infections, with the highest prevalence in developing regions [126, 127].

As previously stated, HPV is the etiological agent of CC, one of the most common gynaecological neoplasia diagnosed worldwide and the leading cause of cancer deaths among women [62, 126]. Despite its widespread global distribution, CC is more commonly diagnosed in underdeveloped and developing regions in Sub-Saharan Africa, South America and Southern Asia. It represents the most common cause of cancer-related death among women in these regions, contrary to developed countries [115, 128]. In addition to CC, HPV is also related to 70% of oropharyngeal carcinomas and other anogenital tumours [64]. Despite mainly resulting from HPV persistent infection, co-factors including cigarette smoking, long-term oral contraceptive use, high parity and co-infection with type 2 herpes simplex virus (HSV-2) or HIV infection are needed for carcinogenesis [10, 129].

To our knowledge, data regarding a direct association between HPV and thromboembolism is lacking (Fig. 3). Nevertheless, some studies point to a putative occurrence of cardiovascular diseases following quadrivalent HPV vaccination [130–133]. Indeed, Gee et al. [134] recognized a tendency for thrombosis occurrence after HPV immunization, despite the lack of statistical significance and the presence of other VTE risk factors. In opposition, other researchers have found no association between both conditions [130–132]. For instance, in a study conducted by Liu et al. [130], whose main objective was to describe the frequencies of adverse events following HPV vaccination, three cases of VTE were observed, however, those women had other health conditions associated with thrombotic risk that could influence the occurrence of such events. Additionally, in the studies performed by Scheller et al. [131] and Yih et al. [132], no association between the quadrivalent HPV vaccine and an increased risk for VTE was observed.

Vaccination implementation was a major step toward preventing infectious diseases and the potential development of viral carcinogenesis, for instance, CC caused by HPV [135]. Generally, vaccines (including the HPV vaccine) are known to induce high and long-lasting immunogenicity, which to some degree depends on the route and site of administration. This immunogenicity is a key factor in determining the strength of the immune response [135, 136]. In the particular case of HPV, contrary to its related vaccine, the virus is known to be poorly immunogenic, not most likely triggering a pro-inflammatory signal with an underlying pro-thrombotic cascade [137]. Several factors influence HPV’s immunogenicity, including viral replication in superficial epithelial cells and HPV’s low or absent systemic viremia (presence in the blood). Overall, the restricted replication combined with its poorly immunogenic nature may explain the absence of an associated thrombo-inflammation in opposition to HPV vaccination. Nevertheless, a potential association between HPV and thrombogenesis cannot be dismissed.

As for VTE incidence in patients with CC, as previously mentioned, data is available in the scientific literature. However, the mechanisms underlying it are not fully defined (Fig. 3).

In conclusion, despite the existing evidence regarding VTE in CC patients, HPV’s direct association with this cardiovascular disease is still unclear. However, it is suggested that HPV vaccination could be a VTE-triggering factor due to its ability to cause a major inflammatory stimulus with a plausible repercussion in haemostasis towards thrombogenesis.

#### Kaposi’s sarcoma-associated herpesvirus (KSHV): Kaposi sarcoma and non-hodgkin lymphoma

KSHV, also called Human Herpesvirus-8 (HHV-8), is an oncovirus that, like EBV, is a member of the *Herpesviridae* family [138]. Apart from causing KS, this pathogen has also been associated with lymphoproliferative disorders, such as NHL, more specifically, a wide spectrum of B-cell lymphomas [139]. Currently, the prevalence of KSHV infection varies according to region, as it is estimated to be lower in the US (1 to 5%) and higher in sub-Saharan Africa (30 to 80%) [140].

As previously mentioned, KS is a rare malignancy, much less common than the infection from which it arises. Although several forms of KS exist, including classic, endemic, iatrogenic and AIDS-related, KSHV is etiologically associated with every KS type [141]. Indeed, nowadays, it is known that KS results from a combination of KSHV infection and immunodeficiency, in addition to other risk factors, therefore being most commonly diagnosed among HIV/AIDS patients [141, 142].

As for KSHV-associated thrombosis, the mechanisms by which the virus may trigger thrombosis have not been described yet (Fig. 3). However, KSHV infection may play a key role in the setting of thrombotic events since the virus preferentially infects endothelial cells, already known to be involved in blood clotting and platelet adhesion [121]. In line, although not fully understood, during KSHV infection, the expression of several host genes involved in apoptosis, angiogenesis, proliferation and inflammation is affected by viral proteins [121]. Consequently, many signalling pathways are activated, including those involved in VEGF production and up-regulation, whose levels have been reported to be increased in thrombosis [143–145]. Indeed, VEGF may be involved in thrombosis by stimulating the secretion of the pro-coagulant factor TF by endothelial cells [144]. Additionally, the production of certain inflammatory components is also thought to be increased due to KSHV infection, as would be expected [146]. These events together, i.e., increased levels of VEGF, TF, and the pro-inflammatory environment upon KSHV infection, could easily influence haemostasis towards a pro-thrombotic state and promote cancer progression [121, 144, 146].

In summary, as previously stated, to our knowledge, data regarding KS-associated VTE mechanisms are lacking (unknown-risk group; Fig. 3). As for KSHV, although it could trigger thrombogenesis in infected patients, the underlying mechanisms are also unclear.

#### Human T-lymphotropic virus type 1 (HTLV-1): adult T cell leukaemia/lymphoma

HTLV-1 is a strain from the Human T-lymphotropic viruses (HLTVs) that belongs to the *Retroviridae* family [147]. The virus, which was the first identified oncovirus, is associated with a lifelong chronic infection known to be the causative agent of several diseases, such as adult T-cell leukaemia/lymphoma (ATL/ATLL), among other inflammatory disorders [64, 148, 149]. Around 5 to 10 million people with HTLV-1 infection worldwide were accounted for in 2012, however, due to a lack of reliable epidemiological data, this number is thought to be higher [149]. As for its geographic distribution, although globally spread, the highest prevalence is reported in Japan, South America, tropical Africa and the Caribbean Islands, where HTLV-1 is endemic, and in certain areas in Australia, Melanesia and the Middle East [64, 149–151].

Among other malignant diseases caused by HTLV-1 chronic infection, ATL is a rare and highly aggressive T-cell neoplasm that, together with T-cell lymphoma subtypes, represents 10 to 15% of NHL [152]. Indeed, approximately 3 to 5% of infected HTLV-1 patients develop ATL after a long latency period, often immunocompromised with a concurrent HIV infection [147, 150]. According to the Globocan database IARC, 3000 ATL cases were globally diagnosed in 2012, the majority being predominantly males [151, 153]. Despite being characterized by clinical manifestations in the lymph nodes, other organs involved in the gastrointestinal and genitourinary tract and central nervous system are also often affected [154]. The ATL patients generally have a poor prognosis compared with other T-cell lymphoma patients, which can be due to either chemoresistance and immunosuppression associated with ATL forms, or the limited treatment options that are not always widely available [154, 155].

To our knowledge, a direct association between HTLV-1 and thromboembolic events, and the putative underlying mechanisms, have not been described in the literature (Fig. 3). However, Dixon [156] reported the presence of thrombotic thrombocytopenic purpura (TTP) in patients with HTLV-1, and according to a review performed by Araujo et al. [157], venous thrombosis appears to be a major complication of HTLV-associated myelopathy/tropical spastic paraparesis in wheelchair-bound patients, a neurodegenerative disease induced by HTLV-1. Additionally, according to Séne et al. [158], ACAs, which have already been described as being frequently associated with viral infections and known to trigger coagulation and inflammatory signalling, are found to be highly prevalent in the acute phase of HTLV-1 infection. Therefore, this phenomenon could constitute a possible trigger for HLTV-1-associated thrombosis [158, 159]. Furthermore, given the high risk for thrombosis among HIV patients, HTLV-1 patients could also show an increased risk, given that HIV and HTLV-1 are both retroviruses.

As for ATL-associated thrombosis, data is lacking. Nonetheless, given the increased incidence among NHL patients, it would be reasonable to assume a putative thrombogenic risk for ATL patients (unknown-risk group; Fig. 3).

#### Merkel cell polyomavirus (MCPyV): Merkel cell carcinoma

The MCPyV is a double-stranded DNA pathogen belonging to the *Polyomaviridae* family. Infection with MCPyV occurs mainly in North America, Europe and Australia. It constitutes the causative agent of a relatively rare but highly aggressive skin cancer known as Merkel cell carcinoma (MCC), prevalent in over 80% of these tumours [64, 160, 161]. According to Krump et al. [161], globally, 88% of adults are positive for MCPyV-specific antibodies, being, therefore, highly prevalent in the human population.

Despite the relatively low incidence, MCC is highly lethal, being responsible for the death of more than one-third of patients [161, 162]. Although also occurring in healthy individuals, this malignancy often arises in the setting of immunodeficiency. For instance, HIV-positive and AIDS patients have a ten-fold risk increase compared to the immunocompetent subjects [160, 161, 163]. Moreover, besides MCPyV infection, additional risk factors promote MCC development, including radiation exposure and advanced age [161, 162].

Regarding VTE incidence in MCPyV-infected patients, to the best of our knowledge, data is lacking in the scientific literature, and no underlying mechanisms have been proposed (Fig. 3). Some case reports indicated thrombosis occurrence in MCC patients, either before or upon cancer diagnosis, however, neither of the studies suggests a connection between MCPyV, MCC and thromboembolism (unknown-risk group; Fig. 3) [164–166].

## Conclusion

Viruses are well-known pathogenic agents responsible for the direct and indirect development of approximately 10% of human cancers. Several viruses linked to oncogenesis in humans have been identified, including HBV, HCV, EBV, HPV, KSHV, HTLV-1, HIV and MCPyV, which are associated with a vast diversity of malignant diseases. Beyond tumorigenesis, viruses are major inducers of blood coagulability. Indeed, haemostasis is frequently disturbed by the pro-inflammatory environment imposed by viral infections, which supports the idea that a viral infection can trigger thromboembolic events.

Conversely, over the years, the association between cancer and thrombosis has been a matter of discussion among researchers. This cardiovascular disease is commonly diagnosed in patients with malignancy and constitutes their second cause of death after cancer. Indeed, there is a bidirectional and complex interplay between both conditions, with tumour cells interacting with haemostatic components and, in parallel, promoting cancer progression and aggressiveness, which worsens the patients’ clinical outcomes.

Although seemingly evident, the literature has not discussed venous thrombosis in viral-induced tumours. Since cancer patients may harbour the oncovirus for their whole life (e.g., chronic infections), and given the remarkably complex interplay of viral infections with both cancer development and haemostasis, it would be of interest to identify a potential pattern of VTE incidence across viral-driven tumours, as well as the putative underlying mechanisms. Except for HCV and HIV, which are associated with an increased VTE risk with several underlying mechanisms proposed, most viruses (HBV, EBV, HPV, HTLV-1 and KSHV) have only a putative associated VTE risk, and few to no mechanisms currently described. On the other hand, for MCPyV, no association was found. Conversely, among the various tumours that can be induced by oncoviruses, GC and HCC were associated with the highest VTE risk, HL, NHL and CC were included in the intermediate VTE risk, while NPC was included in the lowest VTE risk category. Furthermore, to the best of our knowledge, data regarding KS, ATL and MCC association with venous thrombosis was not available in the literature, therefore, considered unknown. Unfortunately, whether the incidence of thrombosis in cancer patients is influenced by tumour aetiology is unexplored, at least to the best of our knowledge, and it is unclear to what extent the presence of viral infections in oncological patients can induce thrombogenesis, being further studies required to fulfil the current gaps in our knowledge.

Overall, the present review summarized the data concerning VTE risk and the associated mechanisms across several viral-induced tumours. This is relevant given the need to better predict VTE development, allowing for better thromboprophylaxis and ultimately improving the prognosis of patients diagnosed with these tumours. Furthermore, the data exposed can be useful to identify potential therapeutic targets that could improve VTE treatment efficacy and decrease the associated side effects, the major one being haemorrhage, which is more common among oncological patients. Beyond the influence of tumour aetiology on thrombosis development, another point to be explored is whether thromboembolic events may facilitate viral infection, reinfection, and viral carcinogenesis.
